# Academic emotions and perceived learning outcomes in GenAI-supported learning: the serial mediating roles of AI acceptance and learning behaviors

**DOI:** 10.3389/fpsyg.2026.1921828

**Published:** 2026-10-14

**Authors:** Huaruo Chen, Gefan Wang, Zhentao Peng, Wanru Song, Jie Xu

**Affiliations:** 1 School of Education Science, Nanjing University of Posts and Telecommunications, Nanjing, Jiangsu, China; 2 School of Education Science, Taizhou University, Taizhou, Jiangsu, China

**Keywords:** academic emotions, AI acceptance, GenAI-supported learning, learning behaviors, learning outcomes, serial mediation

## Abstract

**Objective:**

With the widespread application of generative artificial intelligence (GenAI) in higher education, academic emotions play a vital role in students’ learning processes. This study aims to examine the associations between positive and negative academic emotions and college students’ perceived learning outcomes, and analyze the serial mediating roles of AI acceptance and learning behaviors within GenAI-supported learning environments.

**Methods:**

A total of 526 college students with GenAI learning experience were recruited via convenience sampling. All participants completed four standardized scales measuring academic emotions, AI acceptance, learning behaviors and perceived learning outcomes. Descriptive statistics, correlation analysis, confirmatory factor analysis and bias-corrected Bootstrap mediation tests were conducted using SPSS 26.0 and AMOS 27.0.

**Results:**

All variables were significantly correlated (*p* < 0.01). Positive academic emotions exerted a significant direct association with perceived learning outcomes (*β* = 0.176), whereas the direct association of negative academic emotions was non-significant (*β* = −0.098, *p* = 0.049, 95% CI [−0.205, 0.007]). AI acceptance and learning behaviors had significant independent mediating effects. The serial mediation effect value was 0.020 for positive emotions and −0.037 for negative emotions, with all 95% confidence intervals excluding zero. Indirect pathways dominated the influence of negative academic emotions.

**Conclusion:**

This study identifies a pattern of associations consistent with the hypothesized emotion-cognition-behavior-outcome pathway in GenAI-supported learning. The findings offer empirical evidence for developing targeted academic-emotion-informed intervention strategies, improving students’ AI acceptance and learning engagement, and promoting the high-quality development of intelligent education.

## 1 Introduction

With the rapid development of generative artificial intelligence (GenAI) technology, artificial intelligence tools such as ChatGPT and intelligent tutoring system have been deeply integrated into the whole process of higher education and teaching, which has pushed blended learning into a new intelligent stage (Adeshola and Adepoju, 2024; Chen A. et al., 2026). Leveraging its strengths in interactivity, adaptability and content generation, GenAI provides personalized learning support for college students. Meanwhile, it also brings new challenges related to emotion regulation and technology adaptation (Saracini et al., 2025). As core psychological factors affecting the learning process and outcomes, academic emotions present more complex manifestations in GenAI-enabled environments (Farangi et al., 2025). For instance, positive emotions such as pride and a sense of accomplishment can be intensified by technological empowerment, whereas negative emotions including anxiety and frustration may arise due to barriers in human-computer interaction (Kohnke and Moorhouse, 2025; Wang et al., 2026).

Although GenAI chatbots have been increasingly adopted for instructional writing, their outputs are predominantly presented in plain text, which may impose heavy cognitive load and trigger adverse emotional reactions (Zou et al., 2025). This phenomenon is particularly prominent in second language acquisition and writing tasks (Derakhshan and Wei, 2026). In addition, existing studies have demonstrated that cognition, behaviors and emotions are closely interconnected in human-AI collaborative learning (Edwards et al., 2025). In the specific context of GenAI application, technology acceptance serves as a critical link connecting emotions and behaviors (Chen H. et al., 2026). Students’ perceptions of the usefulness and ease of use of GenAI tools can moderate the transformation from academic emotions to learning behaviors. Positive emotions can enhance students’ AI acceptance and further motivate them to engage in in-depth learning with GenAI (Zhang et al., 2021). By contrast, perceived operational complexity and high energy consumption may form psychological barriers, triggering anxiety, frustration and initial resistance toward relevant technologies (Pavone et al., 2023). Negative emotions tend to reduce AI acceptance willingness and ultimately undermine overall learning performance (Espinoza-Bravo et al., 2025). Nevertheless, extant research has not systematically explored the relationships among academic emotions, AI acceptance, learning behaviors and learning outcomes in GenAI-supported scenarios, nor has it conducted in-depth analysis on the differentiated influencing pathways of distinct academic emotions.

Drawing on the Technology Acceptance Model (TAM) and the Control-Value Theory (CVT), this study constructed a serial mediation model to address the following research questions:

*Question 1*: How do college students’ academic emotions affect learning outcomes in GenAI-supported learning environments?

*Question 2*: Do AI acceptance and learning behaviors play independent mediating roles between academic emotions and learning outcomes?

*Question 3*: Do AI acceptance and learning behaviors exert a serial mediating effect on the relationship between academic emotions and learning outcomes?

*Question 4*: What are the differences in the influencing pathways of positive and negative academic emotions within the overall model?

## 2 Literature review and research hypotheses

### 2.1 Theoretical foundations

This study is grounded in two core theories: the Control-Value Theory (CVT) and the Technology Acceptance Model (TAM). The CVT was proposed by Pekrun (2006), which posits that academic emotions arise from individuals’ perceptions of control over academic tasks and their value judgments. Academic emotions can affect cognitive resource allocation, learning motivation and self-regulation capabilities, and further exert direct and indirect impacts on learning outcomes. In GenAI-supported learning environments, students’ proficiency in operating technical tools and recognition of their learning value can facilitate the development of self-regulated learning abilities, shape academic emotional experiences, and sequentially influence subsequent learning behaviors and outcomes. This provides a fundamental theoretical basis for exploring the relationship between academic emotions and learning outcomes (Mirriahi et al., 2025).

The TAM was developed by Davis (1989), which proposes that users’ acceptance of technology is mainly determined by perceived usefulness and perceived ease of use, which in turn influence usage intention and actual behaviors. Venkatesh and Davis (2000) extended the TAM and verified the predictive effects of perceived usefulness and perceived ease of use on technology usage intention and behaviors. In GenAI application scenarios, TAM acts as a vital theoretical bridge linking academic emotions and learning behaviors (Li et al., 2026). Academic emotions can shape students’ perceptions of the usefulness and ease of use of GenAI tools (i.e., AI acceptance), while AI acceptance can drive the generation and deepening of learning behaviors, and ultimately affect learning outcomes. In addition, Chen H. et al. (2026) developed a learning behavior scale for blended learning based on TAM and confirmed the close association between technology acceptance and various dimensions of learning behaviors, which provides empirical support for the path relationship in the present model.

However, CVT solely elaborates how academic emotions alter learners’ motivational and cognitive processing, without delineating the psychological mechanisms translating affective experience into learners’ evaluative judgments of GenAI tools. To address this theoretical gap, TAM serves as a complementary analytical framework that explicates individual cognitive appraisals of technological artifacts and their subsequent usage behaviors.

The affective mechanisms proposed by CVT and the technology judgment logic embedded in TAM are theoretically interconnected through learners’ real-world GenAI learning experiences. Per broaden-and-build theory, positive academic emotions expand individuals’ cognitive breadth and foster openness toward emerging digital learning instruments, which elevates their perceived usefulness and ease of GenAI systems (Fredrickson, 2001). Conversely, negative academic emotions narrow cognitive focus and amplify perceived operational risks, diminishing learners’ favorable appraisals of AI tools (Beaudry and Pinsonneault, 2010). This two-stage logical chain establishes affective valence (rooted in CVT) as a foundational antecedent to the core TAM constructs of perceived usefulness and perceived ease of use, which sequentially predict learners’ sustained learning engagement.

Consistent with this theoretical linkage, the present serial mediation framework follows the pathway: academic emotions → AI acceptance → learning behaviors → perceived learning outcomes. It should be acknowledged that this study does not incorporate direct measurements of the core control and value appraisal constructs central to CVT, nor does it include additional exogenous predictors featured in extended TAM variants. The integrated model presented herein constitutes a parsimonious theoretical abstraction, and follow-up research with full-scale measurement of all original latent variables from both theories is required to replicate and extend the present findings.

### 2.2 The relationship between academic emotions and learning outcomes

The CVT suggests that academic emotions have valence-specific effects on learning (Pekrun, 2006). Positive academic emotions correlate with expanded cognitive breadth among learners, improve attention distribution and learning motivation, and promote knowledge construction and ability development (Dimitropoulou et al., 2025). By contrast, negative emotions including anxiety and boredom narrow cognitive breadth, increase cognitive load, reduce learning engagement and efficiency, and hinder the achievement of learning goals (Hawthorne et al., 2025). The current study operationalizes academic emotions along the valence dimension rather than distinguishing discrete achievement emotions, which aligns with the core tenets of CVT (Pekrun, 2006). The theory highlights emotional valence as the primary mechanism through which academic emotions reshape cognitive resource allocation and learning motivation, which further determines learners’ judgments of GenAI’s perceived usefulness and ease of use as framed by the TAM. Aggregating discrete achievement emotions into broad positive and negative composite constructs represents a well-established analytical strategy for exploratory educational psychology research (Loderer et al., 2020; Pekrun et al., 2011). Although the GenAI-adapted academic emotion scale deployed in this research has not yet been validated to differentiate granular discrete emotions among Chinese student samples, preliminary factor analysis of the present dataset yields a stable two-factor structure with satisfactory internal consistency (Cronbach’s *α* = 0.936 for negative academic emotions). While the intelligence and interactivity of GenAI create new opportunities for emotion regulation, the core mechanism of how emotional valence affects learning outcomes remains unchanged in intelligent learning environments. Positive emotions may be associated with more effective learning process, whereas negative emotions may be amplified by technology adaptation pressure and associated with adverse impacts on learning. Therefore, the following hypotheses are proposed:

*H1*: Positive academic emotions are expected to be positively associated with perceived learning outcomes within GenAI-supported learning contexts.

*H2*: Negative academic emotions are expected to be negatively associated with perceived learning outcomes in GenAI-supported learning.

### 2.3 Mediating roles of AI acceptance and learning behaviors

#### 2.3.1 The relationship between academic emotions and AI acceptance

In accordance with the CVT, emotional experiences significantly influence individuals’ cognitive judgments of technical tools (Pekrun, 2006). Positive academic emotions can enhance students’ willingness to explore GenAI and their confidence in tool operation, helping them fully recognize the value of GenAI in improving learning efficiency and enriching learning resources, as well as increasing their perceived ease of use (Palalas et al., 2025; Teng, 2026). This finding is also supported by previous studies on teachers’ emotional experiences in AI contexts (Farangi and Nejadghanbar, 2026). Negative academic emotions such as anxiety and frustration amplify concerns about technical risks, reduce recognition of tool value, and lead to avoidance of technical operations, thereby lowering perceived ease of use (Espinoza-Bravo et al., 2025). Therefore, the following hypotheses are proposed:

*H3*: Positive academic emotions are expected to be positively associated with college students’ AI acceptance.

*H4*: Negative academic emotions are expected to be negatively associated with college students’ AI acceptance.

#### 2.3.2 The relationship between AI acceptance and learning behaviors

TAM indicates that technology acceptance is a core predictor of actual usage behaviors (Davis, 1989). In GenAI-supported learning, students with higher perceived usefulness can clearly identify the learning value of GenAI and take the initiative to apply relevant tools for in-depth learning (Liu et al., 2025). Perceived ease of use reduces operational barriers and frustration, and thus promotes the sustainability and depth of learning behaviors (Chen H. et al., 2026). Yang and Ogata (2023) also verified that technology acceptance significantly predicts behavioral, affective and cognitive engagement in learning. Therefore, the following hypotheses are proposed:

*H5*: AI acceptance is expected to be positively associated with learning behaviors in GenAI-supported learning.

#### 2.3.3 The relationship between learning behaviors and learning outcomes

Learning behaviors refer to individuals’ cognitive, affective and behavioral engagement to achieve learning goals, and serve as a critical mediator connecting psychological states and learning outcomes (Fredericks et al., 2004). In GenAI-enabled environments, active learning behaviors with AI tools can effectively raise learning efficiency, facilitate knowledge acquisition and competence development, and further improve learning outcomes (Chen H. et al., 2026). Therefore, the following hypotheses are proposed:

*H6*: Learning behaviors are expected to be positively associated with perceived learning outcomes in GenAI-supported learning.

#### 2.3.4 Serial mediation hypotheses

The influence of academic emotions on learning outcomes is not limited to direct paths. Instead, it may operate through a sequential process in which academic emotions relate to AI acceptance, which in turn is associated with learning behaviors. On the one hand, academic emotions affect learning behaviors by changing AI acceptance, and further influence learning outcomes. On the other hand, academic emotions may directly shape learning behaviors, or indirectly affect learning outcomes through AI acceptance. In GenAI application scenarios, AI acceptance acts as a key intermediate link between emotions and behaviors, making the serial mediating pathway particularly important. Therefore, the following hypotheses are proposed:

*H7*: AI acceptance is expected to mediate the association between positive academic emotions and perceived learning outcomes.

*H8*: AI acceptance is expected to mediate the association between negative academic emotions and perceived learning outcomes.

*H9*: Learning behaviors are expected to mediate the association between positive academic emotions and perceived learning outcomes.

*H10*: Learning behaviors are expected to mediate the association between negative academic emotions and perceived learning outcomes.

*H11*: AI acceptance and learning behaviors will jointly exert a serial mediating effect on the linkage between positive academic emotions and perceived learning outcomes.

*H12*: AI acceptance and learning behaviors will jointly exert a serial mediating effect on the linkage between negative academic emotions and perceived learning outcomes.

### 2.4 Research hypothesis model

This study constructed a serial mediation model, with positive and negative academic emotions as independent variables, learning outcomes as the dependent variable, and AI acceptance and learning behaviors as serial mediators. The direct effects of academic emotions on learning outcomes, as well as the relationships among all variables were examined (see Figure 1).

**Figure 1:**
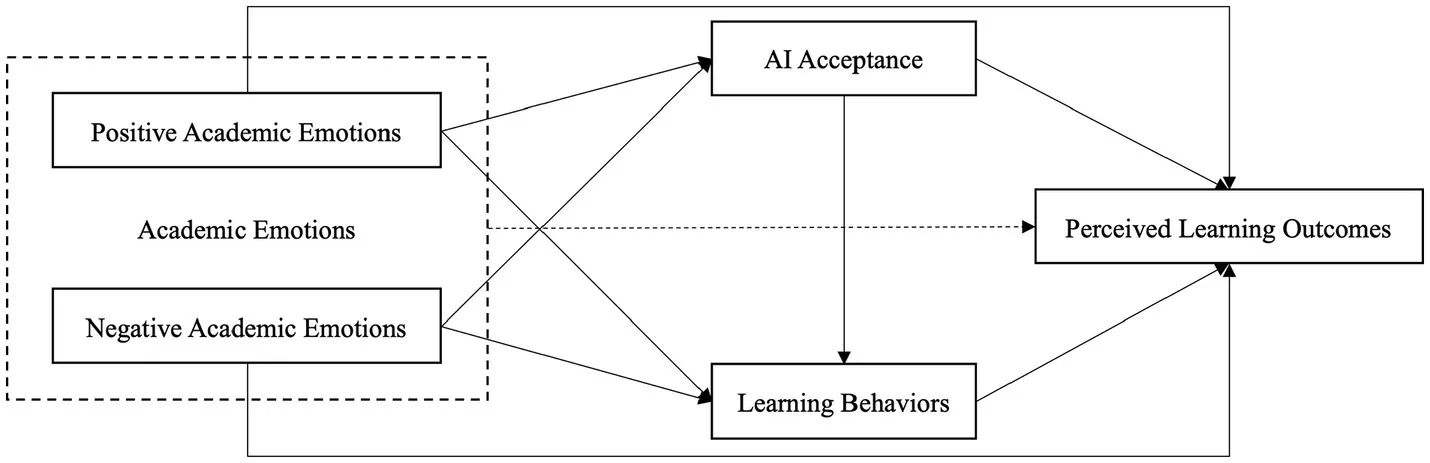
Serial mediation model of academic emotions, AI acceptance, learning behaviors and perceived learning outcomes.

## 3 Methods

### 3.1 Participants

This study adopted convenience sampling and distributed online questionnaires via Wenjuanxing from September to November 2025. All participants were college students with GenAI learning experience. Prior to questionnaire completion, the research purpose and data usage specifications were fully explained, and questionnaire submission was regarded as voluntary participation. Trained research assistants supervised the data collection to guarantee questionnaire quality.

A total of 586 questionnaires were collected. After screening invalid responses (short completion time, regular/identical answers, large-scale missing items and logical contradictions), 526 valid questionnaires were retained, with an effective response rate of 89.76%. The demographic characteristics of participants are presented in Table 1.

**Table 1:** Demographic characteristics of participants (*N* = 526).

Variable	Category	Number	Percentage (%)
Gender	Male	352	66.90%
	Female	174	33.10%
Hometown	Urban	355	67.50%
	Rural	171	32.50%
Major	Natural sciences	443	84.20%
	Humanities and social sciences	83	15.80%
Father’s education	Primary school and below	26	4.90%
	Junior high school	164	31.20%
	Senior high school/Vocational school	161	30.60%
	College/Bachelor’s degree	157	29.80%
	Postgraduate and above	18	3.40%
Mother’s education	Primary school and below	64	12.20%
	Junior high school	170	32.30%
	Senior high school/Vocational school	152	28.90%
	College/Bachelor’s degree	129	24.50%
	Postgraduate and above	11	2.10%

### 3.2 Measurement instruments

All latent variables in this study were assessed using established scales from published literature, appropriately adapted to the AI learning context. All items were rated on a 5-point Likert scale (1 = strongly disagree, 5 = strongly agree).

*Academic Emotion Scale for GenAI Learning* was developed by adapting the Achievement Emotions Questionnaire (Pekrun et al., 2011). The items were translated into Chinese and tailored to the characteristics of GenAI learning contexts before the scale was administered. The scale includes a positive emotions dimension of six items and a negative emotions dimension of six items. Sample items are “I feel curious and excited when learning with GenAI” (positive emotion); “I feel anxious and frustrated when learning with GenAI” (negative emotion). In the present study, Cronbach’s *α* for the positive emotions dimension was 0.900 and for the negative emotions dimension was 0.936, indicating good reliability.

*AI Acceptance Scale* was developed based on TAM and adapted from the study by Abdullah et al. (2016). The items were translated into Chinese and tailored to GenAI learning contexts before the scale was administered. The scale includes a perceived usefulness dimension of four items and a perceived ease of use dimension of four items. Sample items are “GenAI tools can effectively improve my learning efficiency” (perceived usefulness); “I can easily master the operation of GenAI tools” (perceived ease of use). In the present study, the overall Cronbach’s *α* coefficient was 0.903; coefficients for the perceived usefulness and perceived ease of use dimensions were 0.826 and 0.841, respectively, indicating good reliability.

*Learning Behavior Scale for Blended Learning* was developed by referencing the three-dimensional engagement framework of Fredericks et al. (2004) and the Blended Learning Behavior Scale by Chen H. et al. (2026). The items were translated into Chinese and adapted to the GenAI Learning context before the scale was administered. The scale consists of behavioral engagement, emotional engagement, and cognitive engagement dimensions, each comprising four items. Sample items are “I actively participate in GenAI-related online learning activities” for behavioral engagement, “I have a strong interest in learning with GenAI tools” for emotional engagement, and “I use GenAI tools to engage in deep thinking and knowledge integration” for cognitive engagement. In the present study, the overall Cronbach’s alpha for the scale was 0.922, and the alpha coefficients for the behavioral, emotional, and cognitive engagement dimensions were 0.843, 0.816, and 0.785, respectively, indicating good reliability.

*Learning Outcome Scale* was developed based on Bloom’s taxonomy of educational objectives and by adapting the College Students’ Online-Offline Blended Learning Effectiveness Scale developed by Wang et al. (2021). The items were translated into Chinese and adapted to the GenAI learning context before the scale was administered. The scale consists of knowledge mastery, ability improvement, and affective attitude dimensions, each comprising two items, and it employs a 5-point Likert scale with anchors ranging from 1 for strongly disagree to 5 for strongly agree. Sample items are “Through GenAI-supported learning, I have mastered the core knowledge of the course” for knowledge mastery, and “Through GenAI-supported learning, my problem-solving ability has improved” for ability improvement. In the present study, the overall Cronbach’s alpha for the scale was 0.920, and the alpha coefficients for the knowledge mastery, ability improvement, and affective attitude dimensions were 0.852, 0.735, and 0.764, respectively, indicating good reliability. It should be noted that the present learning outcome scale only captures students’ subjective self-perceptions of learning gains rather than objective academic performance metrics such as examination scores or standardized test results. Throughout this manuscript, the term “learning outcomes” exclusively refers to perceived learning effectiveness as reported by participants.

### 3.3 Data analysis

SPSS 26.0 was used for descriptive statistics, correlation analysis and common method bias test. AMOS 27.0 was adopted for confirmatory factor analysis and structural equation modeling. The bias-corrected Bootstrap method (5,000 resamples, 95% confidence interval) was used to test mediating effects. A mediating effect was regarded as significant if the 95% CI did not contain zero.

## 4 Results

### 4.1 Common method bias test

Harman’s single-factor test was conducted for common method bias. The unrotated exploratory factor analysis showed that the model fit was poor (*χ*^2^ = 5628.296, d*f* = 665, *χ*^2^/d*f* = 8.464, GFI = 0.483, AGFI = 0.424, NFI = 0.646, CFI = 0.673, RMSEA = 0.119, SRMR = 0.094), indicating no serious common method bias in this study (Podsakoff et al., 2003).

### 4.2 Descriptive statistics and correlation analysis

As shown in Table 2, the mean scores of all variables ranged from 3.764 to 3.984 (5-point scale), suggesting a moderately high level of positive academic emotions, AI acceptance, learning behaviors and learning outcomes among participants. The mean score of negative academic emotions was 1.926, at a relatively low level. Correlation analysis revealed significant positive correlations between positive academic emotions and AI acceptance, learning behaviors, learning outcomes (*p* < 0.01). Negative academic emotions were significantly negatively correlated with the other three variables (*p* < 0.01). AI acceptance was positively associated with learning behaviors and learning outcomes (*p* < 0.01), and learning behaviors were positively correlated with learning outcomes (*p* < 0.01). The correlation patterns were consistent with theoretical expectations.

**Table 2:** Descriptive statistics and correlation analysis (*N* = 526).

Variable	*M*	SD	Skewness	Kurtosis	1	2	3	4	5
1. Positive academic emotions	3.984	0.633	−0.157	0.239	1				
2. Negative academic emotions	1.926	0.617	0.369	0.658	−0.875**	1			
3. AI acceptance	3.776	0.650	−0.071	0.112	0.634**	−0.648**	1		
4. Learning behaviors	3.865	0.623	0.060	−0.299	0.629**	−0.602**	0.533**	1	
5. Learning outcomes	3.764	0.712	−0.119	0.244	0.638**	−0.610**	0.742**	0.576**	1

**p < 0.01.

### 4.3 Confirmatory factor analysis (CFA)

Confirmatory factor analysis (CFA) was conducted on the five hypothesized latent constructs. The initial unmodified five-factor measurement model exhibited suboptimal fit indices. Based on theoretical rationales and modification indices generated by AMOS, one residual covariance was added between two items within the positive academic emotions dimension that share highly similar wording concerning cognitive curiosity toward GenAI learning (Item PAE1 and Item PAE2). Residual correlations among conceptually homogeneous items from the same latent factor represent a standard, theoretically defensible adjustment in structural equation modeling (Byrne, 2010), and this revision does not introduce cross-dimensional error covariances that would compromise the theoretical structure of the measurement model. Following this targeted adjustment, the model fit improved markedly: *χ*^2^ = 603.904, d*f* = 159, *χ*^2^/d*f* = 3.798, GFI = 0.894, AGFI = 0.860, NFI = 0.938, CFI = 0.953, RMSEA = 0.073, SRMR = 0.049. Consistent with widely accepted SEM evaluation criteria, the CFI, NFI, RMSEA and SRMR all met rigorous cutoffs for acceptable model fit (Hu and Bentler, 1999). By contrast, GFI and AGFI fell marginally below the conventional 0.90 threshold. Prior methodological research has demonstrated that GFI and AGFI are highly sensitive to sample size, model complexity and the number of observed indicators, such that values above 0.85 still signal adequate model performance (Marsh et al., 2004). Per the comprehensive multi-index evaluation framework recommended by Kline (2023), the overall measurement model was judged sufficiently robust to proceed with structural path testing. All standardized factor loadings ranged from 0.684 to 0.954. Each latent dimension yielded composite reliability (CR) greater than 0.80 and average variance extracted (AVE exceeding 0.50), which satisfies the standard thresholds for convergent and discriminant validity (see Table 3).

**Table 3:** Results of confirmatory factor analysis.

Variable	Number of items	Standardized loading	CR	AVE
Positive academic emotions	6	0.684–0.857	0.891	0.579
Negative academic emotions	6	0.805–0.884	0.940	0.723
AI acceptance	8	0.882–0.892	0.881	0.787
Learning behaviors	12	0.797–0.954	0.907	0.766
Learning outcomes	6	0.879–0.918	0.925	0.804

### 4.4 Mediation effect analysis

After controlling for demographic variables (gender, major and GenAI usage duration), the overall model showed good fit. The results of Bootstrap mediation analysis (5,000 resamples) are presented in Table 4.

**Table 4:** Results of mediation effect test.

Path	Effect size	Boot SE	95% bootstrap confidence interval	
			LLCI	ULCI
Positive academic emotion model				
Direct effect (PAE → LO)	0.176	0.071	0.036	0.311
Path 1 (PAE → AIA → LO)	0.485	0.065	0.368	0.625
Path 2 (PAE → LB → LO)	0.082	0.035	0.027	0.164
Path 3 (PAE → AIA → LB → LO)	0.020	0.011	0.003	0.047
Total indirect effect	0.588	0.068	0.467	0.733
Negative academic emotion model				
Direct effect (NAE → LO)	−0.098	0.054	−0.205	0.007
Path 1 (NAE → AIA → LO)	−0.509	0.064	−0.648	−0.399
Path 2 (NAE → LB → LO)	−0.085	0.032	−0.161	−0.035
Path 3 (NAE → AIA → LB → LO)	−0.037	0.014	−0.071	−0.015
Total indirect effect	−0.631	0.061	−0.763	−0.527

PAE, positive academic emotion; NAE, negative academic emotion; LO, learning outcomes; AIA, AI acceptance; LB, learning behaviors.

The results confirmed that AI acceptance and learning behaviors exerted both independent mediating effects and serial mediating effects between academic emotions and learning outcomes. Positive academic emotions were associated with higher perceived learning outcomes via direct effects and multiple indirect pathways; negative academic emotions were predominantly associated with lower perceived learning outcomes through indirect transmission pathways. For negative academic emotions, the direct effect on perceived learning outcomes was small and failed to reach significance based on the bias-corrected Bootstrap confidence interval (*β* = −0.098, 95% CI [−0.205, 0.007]), although the maximum likelihood estimate suggested marginal significance (*p* = 0.048). Given that Bootstrap confidence intervals do not rely on distributional assumptions and are recommended as the primary inferential tool for mediation analysis (MacKinnon et al., 2004; Preacher and Hayes, 2008), this study interpret this direct effect as non-significant. The full standardized path coefficients of the serial mediation model are presented in Figure 2 for visual reference.

**Figure 2:**
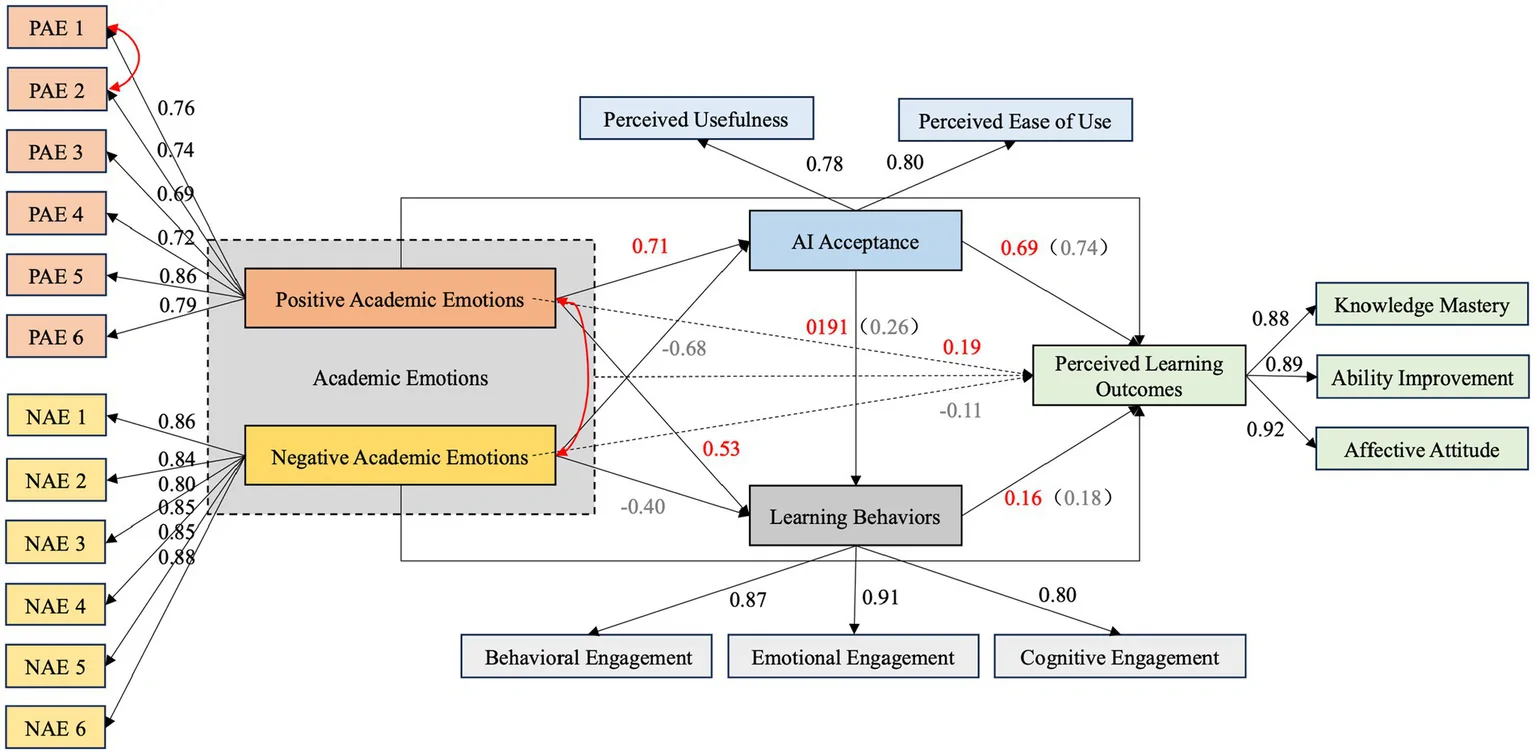
Standardized path coefficients of the mediation model.

## 5 Discussion and conclusions

### 5.1 Discussion

Based on the CVT and TAM, this study constructed a serial mediation model to the associations of positive and negative academic emotions with perceived learning outcomes via AI acceptance and learning behaviors (see Figure 2 for standardized structural paths). The model demonstrated acceptable fit, and the pattern of results was consistent with theoretical expectations.

First, positive academic emotions showed a significant direct association with perceived learning outcomes, while the direct association of negative academic emotions with perceived learning outcomes was not significant. This pattern aligns with the core propositions of the Control-Value Theory, which posits that positive emotions are generally accompanied by more adaptive cognitive allocation and stronger learning motivation, whereas negative emotions tend to co-occur with increased cognitive load and reduced learning engagement. Although GenAI may assist emotion regulation, the valence-specific patterns of association observed in this study are consistent with the view that emotional valence remains an important correlate of perceived learning in intelligent learning environments.

Second, AI acceptance played a significant independent mediating role. Academic emotions were associated with students’ perceptions of GenAI’s usefulness and ease of use, which in turn were associated with learning behaviors and perceived outcomes. This finding is consistent with the applicability of TAM in GenAI learning scenarios. Positive emotions were associated with higher technology recognition and acceptance; negative emotions were associated with resistance and lower perceived utility of AI tools.

Third, learning behaviors also served as an independent mediator. Among behavioral, affective and cognitive engagement, affective engagement showed the strongest predictive power for learning outcomes. In GenAI environments, positive emotions were associated with greater learning interest and intrinsic motivation, which in turn were related to multi-dimensional engagement; negative emotions were associated with lower affective participation and overall learning quality.

Most importantly, the significant serial-mediation effect yields findings consistent with our hypothesized indirect sequence, in which academic emotions relate to AI acceptance, which then links to learning behaviors and further connects to perceived learning outcomes. These results add empirical insights into the interrelations among emotion, technology-related cognition, and learning behaviors within GenAI-supported learning contexts. For negative academic emotions, indirect pathways accounted for the majority of the total association, which suggests that AI acceptance and learning behaviors may serve as important intervening variables in the association between negative emotions and perceived learning outcomes.

The magnitudes of the observed effects warrant careful interpretation against conventional benchmarks (Cohen, 2013). A nuanced picture emerges when direct, specific indirect, and total indirect effects are considered separately.

For positive academic emotions, the direct effect on perceived learning outcomes was small (*β* = 0.176, 95% CI [0.036, 0.311]), indicating that after accounting for AI acceptance and learning behaviors, only a modest direct association remained. The specific indirect effect through AI acceptance alone (Path 1: *β* = 0.485, 95% CI [0.368, 0.625]) was large in magnitude, representing the dominant mechanism by which positive emotions are associated with perceived learning outcomes—more than twice the size of the direct effect. This suggests that the primary way positive emotions relate to learning outcomes in GenAI contexts is not through some direct motivational pathway, but rather through enhanced perceptions of AI tools’ usefulness and ease of use.

For negative academic emotions, the pattern is even more striking. The direct effect was not significant (*β* = −0.098, 95% CI [−0.205, 0.007]), indicating full mediation. The specific indirect effect through AI acceptance (Path 1: *β* = −0.509, 95% CI [−0.648, −0.399]) was the single largest pathway in the entire model, exceeding any other standardized coefficient. This finding suggests that negative emotions’ association with poorer perceived learning outcomes may be largely accounted for by reduced technology acceptance—students who feel anxious or frustrated tend to perceive GenAI tools as less useful and harder to use, and this diminished acceptance, in turn, is strongly associated with lower perceived learning outcomes.

The serial-mediation effects representing the sequential association from academic emotions through AI acceptance and learning behaviors toward perceived learning outcomes were notably small for both positive (*β* = 0.020) and negative (*β* = −0.037) emotions. This attenuation is expected in multi-link mediation chains: each successive pathway compounds standard errors and reduces the specific indirect effect, even when each individual link is substantively meaningful. In the present model, the serial chain depends on three multiplicative paths (emotion → AI acceptance, AI acceptance → learning behaviors, learning behaviors → outcomes), each of which attenuates the final estimate. The small magnitude of the serial chain effects does not indicate that the theoretical sequence is unimportant; rather, it suggests that the practical returns from targeting the complete four-variable chain may be limited compared to interventions focused on the strongest individual link—namely, AI acceptance.

From an educational practice standpoint, even small effects can accumulate meaningful impact when considered across large student populations over extended instructional periods (Kraft, 2020). The total indirect effects (0.588 for positive emotions and −0.631 for negative emotions) are large by conventional standards and represent the aggregate influence flowing through all mediating pathways combined. Moreover, the Path 1 effects (0.485 and −0.509) are not small—they are among the largest standardized coefficients in the model and indicate that AI acceptance is a powerful conduit through which academic emotions are associated with perceived learning outcomes. The practical implication is clear: interventions aimed at improving students’ perceptions of GenAI tools’ usefulness and ease of use may yield greater returns on perceived learning outcomes than interventions targeting emotional states directly or attempting to manipulate the entire emotion–cognition–behavior–outcome chain simultaneously.

### 5.2 Conclusion

Combining the CVT and TAM, this study examined the associations among academic emotions, AI acceptance, learning behaviors and learning outcomes in GenAI-supported learning. Positive academic emotions were directly associated with higher perceived learning outcomes and were also indirectly associated with outcomes through the serial pathway of higher AI acceptance and greater learning engagement. The indirect effect through AI acceptance alone (*β* = 0.485) was substantially larger than the direct effect (*β* = 0.176), underscoring the central mediating role of technology perceptions. Negative academic emotions were associated with lower perceived learning outcomes mostly through indirect pathways (total indirect effect = −0.631), with no significant direct association remaining.

Affective engagement emerged as the dimension most strongly associated with perceived learning quality. Taken together, these findings are consistent with an emotion–cognition–behavior–outcome chain in GenAI learning, while acknowledging that the cross-sectional design precludes causal conclusions.

### 5.3 Practical implications

Based on the patterns of association observed among connecting academic emotions, technology acceptance, learning behavior, and learning effectiveness of this study, and considering the reality that artificial intelligence is deeply integrated into the entire learning process, this study offers systematic practical pathways for optimizing learning experience, enhancing learning efficacy, and promoting deep learning. Practical implications are proposed from four dimensions, namely emotion cultivation, technology empowerment, behavior optimization, and integrated education. These implications provide theoretical foundations and practical references for universities to build intelligent, personalized, and precise learning support systems.

First, universities may consider establishing a system for monitoring and supporting academic emotions. Universities could explore intelligent learning platforms to build mechanisms for real-time identification, dynamic tracking, and targeted intervention of academic emotions that cover the entire learning cycle. Through stratified task design, immediate process-oriented feedback, and visualization of milestone achievements, positive academic emotions may be supported while negative academic emotions such as technology anxiety and learning burnout may be mitigated, thereby laying a stable emotional foundation for the deep integration of artificial intelligence into the learning process. Meanwhile, universities may consider providing students with emotion regulation and stress management training to enhance their emotional awareness, emotional expression, and emotional regulation abilities in intelligent learning environments, forming a synergistic pattern of external support and internal regulation for emotion cultivation.

Second, the usability and value perception of AI learning tools may be strengthened to enhance students’ technology acceptance. Universities could consider offering functional explanations, operational training, and application demonstrations of AI learning tools to lower students’ technical barriers and cognitive load, thereby enhancing their perceived usefulness, perceived ease of use, and intention to use AI tools, so that technology truly serves as an “empowering tool” for learning rather than a “cognitive burden.” By sharing typical cases, facilitating peer experience exchanges, and providing teacher demonstrations and guidance, universities may help strengthen students’ recognition of the value of artificial intelligence in improving learning efficiency, expanding learning resources, and optimizing learning pathways, thereby shifting students from “passive use” to “active acceptance and deep application.”

Third, high-quality engagement in learning behaviors may be prioritized to promote deep learning. Based on the characteristics of AI-enabled learning environments, universities could design stratified and classified learning tasks that combine interest, interactivity, inquiry, and challenge, enhancing the alignment of these tasks with students’ interests, professional needs, and ability levels. Particular attention may be directed toward improving students’ emotional engagement, behavioral engagement, and cognitive engagement in the learning process, with particular emphasis on strengthening the driving role of emotional engagement in learning behaviors. This may support a shift from superficial “task completion” to deep meaning construction, reflective critique, and innovative practice, thereby forming a high-quality learning loop.

Fourth, the effective implementation of an integrated “emotion-cognition-behavior” educational model may be advanced. Universities could explore incorporating the cultivation of positive emotions, the enhancement of technology acceptance, and the optimization of learning behaviors into an integrated education system, breaking down the barriers among psychological support, technology training, and academic guidance, and building a synergistic mechanism that deeply integrates psychological empowerment, technological empowerment, and academic empowerment. By integrating the efforts of academic advisors, subject instructors, lab instructors, IT personnel, and other stakeholders, a comprehensive support system may be developed that covers the entire process from pre-class, in-class, to post-class and spans both online and offline settings. This may help realize the systemic empowering value of artificial intelligence in higher education and support students’ adaptability, learning efficacy, and developmental potential in intelligent learning environments.

### 5.4 Limitations and future research

This study has several limitations. First, this study adopted a cross-sectional design, which precludes causal inference. Longitudinal tracking or experimental designs are recommended for follow-up research. Second, the sample was limited to undergraduate students at regular universities; future research can expand samples to postgraduates and students from different disciplines to improve external validity. Third, all indicators of learning outcomes rely solely on self-reported subjective evaluations rather than objective achievement data. Prior meta-analytic evidence demonstrates a substantial divergence between self-rated learning gains and actual academic performance (Sitzmann et al., 2010). Affective states such as positive academic emotions may inflate learners’ subjective judgments of their progress independent of real knowledge acquisition or skill improvement. Subsequent research could integrate objective assessment indicators (e.g., course exam grades, post-learning knowledge tests) to conduct cross-validation and improve measurement validity. Fourth, although the modified measurement model demonstrated generally acceptable fit (CFI = 0.953, RMSEA = 0.073, SRMR = 0.049), the GFI (0.894) and AGFI (0.860) remained slightly below the conventional 0.90 threshold. These values may reflect residual cross-loadings among the three engagement sub-dimensions of the Learning Behavior Scale or item-level wording effects in the Chinese adaptations. Future research should consider testing alternative factor structures to further improve measurement precision. Fifth, potential moderators such as digital literacy and teacher support were not included. Sixth, this study did not distinguish different types of GenAI tools. Future research could conduct comparative studies on different tools such as intelligent tutoring, intelligent assessment, and collaborative writing, thereby providing more targeted evidence for the precise application of artificial intelligence in educational settings.

Future research can carry out in-depth exploration on the neural mechanisms of academic emotions, cross-cultural comparisons, and adaptive intervention design based on GenAI systems to continuously deepen the understanding of learning patterns in the era of artificial intelligence and provide more systematic, scientific, and actionable theoretical support and practical guidance for intelligent education reform.

## Data Availability

The raw data supporting the conclusions of this article will be made available by the authors, without undue reservation.
